# Supplementary material for: Foundation models enable wearable signal screening for cardiovascular disease among people living with HIV
Source: Commun Med (Lond). 2026 Jan 16;6:72. doi: 10.1038/s43856-025-01331-6 (PMC12868744; doi:10.1038/s43856-025-01331-6)
Supplement: Supplementary file 6 — Supplementary Information [file 43856_2025_1331_MOESM6_ESM.pdf]

1 Data

1.1 Study Population and Setting

This study enrolled 80 asymptomatic adults living with HIV (PLWH) attending the outpatient department of the Hospital for Tropical Diseases (HTD) in Ho Chi Minh City, Vietnam, between 17th November 2023 and 20th July 2024. Eligibility criteria included age ≥18 years and stable HIV treatment. Patients with acute illness or current hospitalisation were excluded. Written informed consent was obtained from all participants. The study was approved by the HTD Ethics Committee and the Oxford Tropical Research Ethics Committee.

Participants were followed up over four outpatient visits across a 12-month period. All 80 patients attended Visit 2, 79 attended Visit 3, and 74 attended Visit 4. At each visit, participants underwent wearable monitoring, ECG, and a brief acceptability survey.

1.2 Wearable Signal Acquisition and Feature Extraction

PPG waveforms were collected using SmartCare wearable pulse oximeters (SmartCare Analytics UK), transmitting data via Bluetooth at 100 Hz to an Android mobile application ("SmartCare Capture"). Each participant was monitored for approximately 20 minutes. After quality control (noise and flatline detection), over 95% of signal data were retained. From these recordings, five-minute high-quality PPG segments were selected for each patient and used for physiological feature extraction.

Figure 1 shows the complete SmartCare BM2000A pulse oximeter setup used for PPG data acquisition. The device kit included: (1) SmartCare watch with OLED display, (2) adjustable wrist strap, (3) finger sensor cable with red/infrared LEDs, (4) USB charging cable, and (5) Samsung tablet with SmartCare Capture application for real-time visualisation and data storage.

Participants were seated comfortably in the outpatient waiting area. The following standardised protocol was followed:

1. Attach the finger sensor cable to the SmartCare watch main unit
2. Secure the device to the participant's non-dominant wrist using the adjustable strap
3. Position the finger sensor on the index or middle finger with the nail symbol facing upward
4. Power on the device and verify red sensor light activation
5. Confirm Bluetooth connection to the paired tablet (within 5-10 meters)
6. Monitor real-time signal quality via SmartCare Capture application
7. Record continuously for 20 minutes, with the first 15 minutes used for analysis

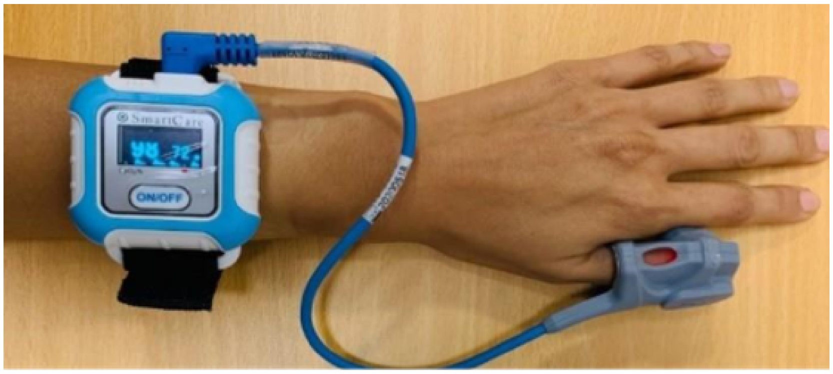

Figure 1: SmartCare wearable pulse oximeter components and patient setup. (a) Complete device kit showing the wrist unit, finger sensor, and data collection tablet. (b) Proper sensor placement with nail-side orientation for optimal signal quality. (c) Real-time PPG waveform display on the SmartCare Capture application during a 20-minute recording session. The CE-marked device transmitted data via Bluetooth at 100 Hz, with quality control achieving >95% usable signal retention across all participants.

8. After completion, disconnect via the app before removing the device

Signal quality was monitored throughout by trained clinic staff. Participants were instructed to remain seated with minimal arm movement during recording. The device automatically filtered motion artifacts and provided real-time feedback on signal quality through the tablet interface.

Heart rate variability (HRV) and PPG morphology features were extracted using the open-source `vital_DSP` and `vital_SQI` Python packages. In total, 61 HRV and waveform morphology features were computed per patient. Selected features include time-domain (e.g., RMSSD, SDNN), frequency-domain (e.g., LF/HF power), non-linear (e.g., Poincaré, DFA), and morphological (e.g., systolic slope, diastolic duration) metrics.

1.3 Clinical Feature Compilation

Baseline clinical and demographic variables were collected, including age, sex, treatment history, smoking status, and systolic/diastolic blood pressure. Additional features were derived from up to four outpatient visits, including total cholesterol, HDL, and systolic blood pressure. Time-varying features were aggregated via mean, delta, slope, min/max, and range. A full list of clinical features used for modelling is summarised in Table 1.

1.4 Cardiovascular Disease Prevalence and Risk Score Distributions

Of the 80 patients, 13 were found to have ECG or echocardiographic evidence of CVD. Risk score distributions were skewed: 65% (52/80) were classified as low-risk by Framingham, compared to 45% (36/80) by D:A:D. Importantly, several CVD-positive patients were misclassified as low-risk by both scores, highlighting the limitations of existing screening tools in this population.

1.5 Summary of Patient Characteristics and Feature Distributions

Table 1 summarises clinical, demographic, and waveform-derived feature distributions across the full cohort of 80 participants. Continuous variables are reported as median (IQR); categorical variables are reported as n (%).

2 Training Details

2.1 Model Implementation and Frameworks

All supervised machine learning models were implemented in Python using `scikit-learn` (v1.3.0) and related packages including `xgboost`, `lightgbm`, and `tabpfn`. Model development, hyperparameter tuning, and evaluation were conducted using reproducible pipelines with fixed seeds (42) for all random splits.

All preprocessing steps were performed using `pandas`, `numpy`, and `scikit-learn` preprocessing modules. Data was split into stratified training and held-out test sets (80/20), with bootstrapping used to estimate performance variability across 10 resamples. Model calibration was conducted using isotonic regression fit on the training set. Experiments were conducted on a MacBook Pro (Apple M3 Pro chip, 51 GB RAM) without GPU acceleration, reflecting the resource-constrained, low-cost settings in which our approach is designed to operate.

2.2 Supervised Model Training and Tuning

The following models were implemented and evaluated:

- Logistic Regression with ElasticNet regularisation (`ElasticNet`)
- Random Forest (`RandomForestClassifier`)
- Light Gradient Boosting Machine (`LightGBM`)
- Extreme Gradient Boosting (`XGBoost`)
- TabPFN (pretrained foundation model)
- Decision Tree (CART)

Table 1: Baseline demographic, clinical, and waveform feature distributions stratified by cardiologist-confirmed CVD status. Values are shown as median (IQR) or count (%). Percentages are column-wise.

Feature	Overall (N=80)	CVD = 1 (N=13)	CVD = 0 (N=67)
**Demographics**			
Age (years)	42 (35, 49)	47 (44, 56)	38 (28, 44)
Sex (Male)	57 (71%)	12 (92%)	45 (67%)
Current smoker	21 (26%)	9 (69%)	12 (18%)
Diabetes	3 (3.8%)	3 (23%)	0 (0%)
Hypertension (treatment)	5 (6.3%)	2 (15%)	3 (4.5%)
Family history of CVD	50 (63%)	10 (77%)	40 (60%)
**Vital Signs and Labs**			
Systolic BP (mmHg)	120 (110, 130)	130 (120, 140)	110 (100, 120)
HDL Cholesterol (mmol/L)	1.09 (0.94, 1.25)	1.00 (0.85, 1.12)	1.11 (0.97, 1.28)
Total Cholesterol (mmol/L)	4.29 (3.83, 4.79)	4.72 (4.15, 5.45)	4.18 (3.75, 4.65)
CD4 Cell Count (cells/mm^3)	510 (391, 623)	445 (320, 575)	525 (410, 640)
**Framingham Score (10yr)**			
Low risk	52 (65%)	5 (38%)	47 (70%)
High risk	28 (35%)	8 (62%)	20 (30%)
**D:A:D Modified Score**			
Low risk	36 (45%)	4 (31%)	32 (48%)
High risk	44 (55%)	9 (69%)	35 (52%)
**HRV Features (PPG-derived)**			
SDNN (ms)	155 (71, 304)	88 (52, 165)	175 (85, 330)
RMSSD (ms)	210 (93, 431)	125 (68, 285)	235 (108, 475)
pNN50 (%)	13 (5, 32)	7 (3, 19)	15 (6, 36)
Mean NN (ms)	818 (746, 921)	780 (710, 865)	830 (760, 940)
Total Power (ms^2)	6,032 (1,496, 16,578)	3,100 (950, 9,800)	7,250 (1,850, 18,500)
LF/HF Ratio	0.69 (0.52, 1.00)	0.92 (0.68, 1.35)	0.65 (0.48, 0.92)
DFA	0.90 (0.84, 0.98)	0.96 (0.89, 1.05)	0.89 (0.83, 0.96)
**PPG Morphology Features**			
Systolic duration (s)	0.43 (0.40, 0.47)	0.42 (0.38, 0.46)	0.43 (0.40, 0.48)
Diastolic duration (s)	0.39 (0.34, 0.45)	0.37 (0.32, 0.42)	0.40 (0.35, 0.46)
Systolic slope	1.55 (1.44, 1.56)	1.54 (1.42, 1.56)	1.55 (1.45, 1.56)
Diastolic slope	-1.563 (-1.568, -1.553)	-1.560 (-1.566, -1.548)	-1.564 (-1.569, -1.555)
Systolic amplitude variability	6,021 (4,776, 6,970)	7,100 (5,450, 8,650)	5,750 (4,600, 6,700)
Heart rate (bpm)	76 (68, 84)	80 (73, 90)	74 (66, 82)

For each model, a three-fold stratified cross-validation strategy was used on the training set for hyperparameter optimisation using Bayesian search via `scikit-optimize`. Performance was evaluated using AUROC, average precision (AP), F1 score, precision, and recall. The final model was refit on the full training set before evaluating on the test set.

2.3 Hyperparameter Search Spaces and Final Selections

ElasticNet:

- `penalty` (L1/L2 mixing ratio): [0.1, 0.5, 0.9]
- `alpha` (regularisation strength): log-uniform $[10^{-4}, 10^{1}]$

Random Forest:

- `n_estimators`: 100, 250, **500**
- `max_depth`: None, 5, 10, 20
- `min_samples_leaf`: 1, 2, **5**
- `class_weight`: 'balanced'

LightGBM:

- `num_leaves`: 15, 31, 63
- `min_data_in_leaf`: 5, 10, 20
- `learning_rate`: 0.1, 0.01, **0.005**
- `n_estimators`: **500**

XGBoost:

- `learning_rate`: 0.01, 0.05, **0.1**
- `max_depth`: 3, **5**, 7
- `subsample`: 0.5, 0.8, 1.0
- `n_estimators`: 100, **300**

TabPFN: used without tuning; we employed the pretrained model provided by the authors on the OpenML default setting with 10,000 budgeted inference steps.

Decision Tree:

- `max_depth`: 3, 5, 7, None
- `criterion`: 'gini', 'entropy'

3 Foundation Model Prompting

To evaluate the zero-shot predictive capability of foundation models for cardiovascular disease (CVD) risk, we designed controlled prompting procedures for NormWear and PaPaGei, each operating in inference-only mode on electrocardiogram (ECG) and photoplethysmography (PPG) signals, respectively.

3.1 NormWear Prompt Design

For NormWear experiments, we used the `NormWearZeroShot` PyTorch implementation, loading pretrained weights and model-specific tokenisers as described in the original release. NormWear accepts paired ECG signals and text prompts, performing multimodal alignment via contrastive inference. We used the following prompt setup:

- **Query:** `"This is a 3-lead ECG from a patient. What does it indicate?"`
- **Options:**
  - `"The patient has no signs of cardiovascular disease."`
  - `"The patient shows signs of cardiovascular disease or stress."`

Each 3-lead ECG was segmented into 2-second windows (128 samples at 64 Hz) and up to 20 high-quality windows per patient were selected using signal quality control filters. Per-window predictions were generated using similarity scores between the ECG embeddings and text embeddings. These predictions were aggregated at the patient level using majority voting and averaged probabilities. No task-specific fine-tuning was performed, and the model operated fully in zero-shot mode.

3.2 PaPaGei Prompt-Free Inference

In contrast, PaPaGei is a general-purpose encoder model producing dense time-series embeddings without explicit prompting. We employed the `ResNet1DMoE` architecture with the following configuration:

- `base_filters = 32`, `kernel_size = 3`, `stride = 2`
- `n_block = 18`, `n_experts = 3`, `embedding dimension = 512`

Each 10-second PPG window (resampled to 100 Hz) was denoised, z-score normalised, and filtered using Vital-SQI criteria to remove poor-quality segments. Embeddings were extracted from the penultimate layer of the pretrained model. For downstream classification:

1. Window-level embeddings were aggregated per patient via mean pooling.
2. Dimensionality reduction was performed via principal component analysis (PCA, top 15 components).
3. A Random Forest classifier was trained on patient-level embeddings using 10 bootstrapped train-test splits.
4. Model calibration was performed using isotonic regression on the training set.

No fine-tuning or additional supervision was applied to PaPaGei during this process, consistent with the zero-shot foundation model paradigm.

3.3 Inference Code Snippets

NormWear prompting setup:

```
task = ["This is a 3-lead ECG from a patient. What does it indicate?"]
options = [
    "The patient has no signs of cardiovascular disease.",
    "The patient shows signs of cardiovascular disease or stress."
]
txt_embed = model.txt_encode(task + options)
query_embed, option_embed = txt_embed[:1], txt_embed[1:]
signal_embed = model.signal_encode(ecg_tensor_batch, query_embed)
probs = model.inference(signal_embed, option_embed)
```

PaPaGei embedding extraction:

```
model = ResNet1DMoE(**model_config).to(device)
model.load_state_dict(torch.load("papagei_s.pt"))
model.eval()
embedding = model(signal_tensor)  # Shape: [batch_size, 512]
```

A complete list of preprocessing steps, model parameters, and inference scripts is available at our code repository [link redacted for peer review].

4 Supplementary Note: Additional Experiments

Supplementary Table 2 contains additional metrics from Table 1 in the main text.

Table 2: Complete classification performance metrics at the selected operating threshold for key models. Values are mean ± SD over 10 bootstrap resamples. Sensitivity (recall) and specificity characterise the model's ability to correctly identify positive and negative cases, respectively. PPV (positive predictive value, equivalent to precision) indicates the proportion of positive predictions that are correct. NPV (negative predictive value) indicates the proportion of negative predictions that are correct. For screening applications, high sensitivity and NPV are prioritized to ensure no cases are missed.

Model	Recall	Specificity	PPV (Precision)	NPV	Acc*	AUPRC
**Clinical only**						
Random Forest	1.000 ± 0.00	0.558 ± 0.16	0.306 ± 0.17	1.000 ± 0.00	0.779 ± 0.08	0.433 ± 0.11
ElasticNet	0.500 ± 0.19	0.902 ± 0.07	0.500 ± 0.17	0.902 ± 0.04	0.701 ± 0.09	0.340 ± 0.13
TabFPN	0.500 ± 0.20	0.916 ± 0.07	0.538 ± 0.21	0.904 ± 0.04	0.708 ± 0.10	0.412 ± 0.15
**PPG-derived representations**						
PCA	0.250 ± 0.12	0.806 ± 0.15	0.300 ± 0.12	0.828 ± 0.03	0.528 ± 0.11	0.208 ± 0.10
NormWear (zero-shot)	0.250 ± 0.12	0.854 ± 0.12	0.333 ± 0.14	0.832 ± 0.03	0.552 ± 0.10	0.226 ± 0.10
**PaPaGei**	**1.000 ± 0.00**	**0.806 ± 0.12**	**0.389 ± 0.16**	**1.000 ± 0.00**	**0.903 ± 0.06**	**0.489 ± 0.12**
Baseline expectation						
Random classifier: Sensitivity = 0.50, Specificity = 0.50, PPV = 0.163 (prevalence), NPV = 0.837, AUPRC = 0.163						

References
